# Social Isolation Among Older Military Veterans

**DOI:** 10.1093/geroni/igab046.2069

**Published:** 2021-12-17

**Authors:** Zainab Suntai, Kirsten Laha-Walsh

**Affiliations:** University of Alabama, Tuscaloosa, Alabama, United States

## Abstract

Social isolation is an increasingly critical issue among older adults and has been found to affect several domains of well-being, including physical, psychological, and cognitive health. Research has found that military veterans often experience hardships in the transition back to civilian life including emotional trauma, depression, substance misuse and pain from combat-related injuries, which have been shown to persist well into older adulthood. As such, this study aimed to examine the prevalence of social isolation among older military veterans and determine which veterans are most at-risk of experiencing social isolation, using the Berkman-Syme Social Network Index as a framework. Data were derived from Round 1 of the National Health and Aging Trends Study (NHATS), an annual longitudinal panel survey of adults aged 65 and older living in the United States. Results showed that about 4.5% of veterans in the NHATS are severely socially isolated while another 20.9% are socially isolated. After controlling for other explanatory variables, being White, being 85 and older, having lower educational attainment, being unmarried/unpartnered and having lower income were associated with an increased risk of experiencing social isolation. Interventions aiming to improve the well-being of older veterans should consider employing both preventative and amendatory measures. These may include the creation and administration of a standardized social isolation scale during visits to veterans’ affairs (VA) medical centers and a general effort to address stressors from military service by destigmatizing and improving access to mental health services.

